# The Temporal Retinal Nerve Fiber Layer Thickness Is the Most Important Optical Coherence Tomography Estimate in Multiple Sclerosis

**DOI:** 10.3389/fneur.2017.00675

**Published:** 2017-12-13

**Authors:** Ulrika Birkeldh, Ali Manouchehrinia, Max Albert Hietala, Jan Hillert, Tomas Olsson, Fredrik Piehl, Ingrid Skelton Kockum, Lou Brundin, Ori Zahavi, Marika Wahlberg-Ramsay, Rune Brautaset, Maria Nilsson

**Affiliations:** ^1^Unit of Optometry, Department of Clinical Neuroscience, St. Erik Eye Hospital, Karolinska Institute, Stockholm, Sweden; ^2^Department of Clinical Neuroscience, Karolinska Institute at Karolinska University Hospital Solna, Stockholm, Sweden

**Keywords:** multiple sclerosis, retinal nerve fiber layer, ganglion cell–inner plexiform layer, optical coherence tomography, cognitive impairment, physical disability

## Abstract

**Background:**

Reduced peripapillary retinal nerve fiber layer (pRNFL) and combined ganglion cell and inner plexiform layer (GCIP) thicknesses as measured by optical coherence tomography (OCT) have been observed in multiple sclerosis (MS) patients. The purpose was to determine the most associative OCT measure to level of cognitive and physical disability in MS.

**Methods:**

Data were collected from 546 MS patients and 175 healthy controls (HCs). We compared the average pRNFL, temporal pRNFL (T-pRNFL), overall inner ganglion cell/inner plexiform layer (GCIP), and the overall ganglion cell complex (GCC) including macular RNFL and GCIP thicknesses measurements in differentiating MS subtypes from HCs. The association between OCT measures, Expanded Disability Status Scale (EDSS), and Symbol Digit Modalities Test (SDMT) were assessed using generalized estimating equations models.

**Results:**

Both peripapillary and macular OCT measurements could differentiate all MS subtypes from HCs. The SDMT score was significantly associated with reduced thickness of all OCT measures, mostly in average pRNFL (0.14 µm, *P* = 0.001) and T-pRNFL (0.17 µm, *P* < 0.001). The EDSS score was significantly associated with reduced inner retinal layer thickness. The largest reduction was seen in T-pRNFL (−1.52 μm, *P* < 0.001) and inner GCC (−1.78 μm, *P* < 0.001).

**Conclusion:**

The T-pRNFL is highly sensitive and associated with level of both cognitive and physical disability.

## Introduction

Multiple sclerosis (MS) is a chronic inflammatory disease of the central nervous system (CNS), associated with development of both cognitive impairment and physical disability. So far MRI-based methods have been the most established method to assess degree of CNS damage. However, a growing body of evidence suggests that also optical coherence tomography (OCT) can be used to estimate degree of neurodegeneration. Reduced peripapillary retinal nerve fiber layer (pRNFL) thickness as measured with OCT has been observed in MS patients with and without history of optic neuritis (ON) (1–6) and in other neurological conditions such as cluster headache (7). The retina is part of the CNS and measurements of the un-myelinated axons in the eye can point toward the overall neural degeneration associated with the disease (8). Some studies have suggested OCT as an important tool for monitoring MS and as a complementary measure to magnetic resonance imaging (MRI) (9–12). Functional and/or cognitive disability have been found to be correlated with pRNFL thickness in some (13–15), but not all studies (9, 16).

Lately, software advancements of the SD-OCT technique allow measurement and automatic segmentation of the ganglion cell–inner plexiform layer (GCIP). Reduced GCIP thickness has been found in MS (8, 17, 18) and also in Parkinson’s disease (19), Alzheimer’s disease (20), Susac’s syndrome (21), idiopathic intracranial hypertension (22), and neuromyelitis optica (23). Furthermore, the GCIP thickness in MS patients has been shown to correlate better than pRNFL thickness with functional outcomes such as visual function and the Expanded Disability Status Scale (EDSS) score (24), the most established measure of physical disability in MS.

Today, there are numerous parameters that could be used to describe the retinal structure. Identification of a single and robust OCT parameter in a large population based study would be beneficial to research studies and in daily clinical work. Hence, the aim of the this study was to identify the most sensitive macular parameter to disability change in MS by comparing different OCT outcomes and their correlation to clinical data such as history of ON, MS subtype, disease duration, EDSS score, and cognitive function.

## Materials and Methods

**Figure 1:**
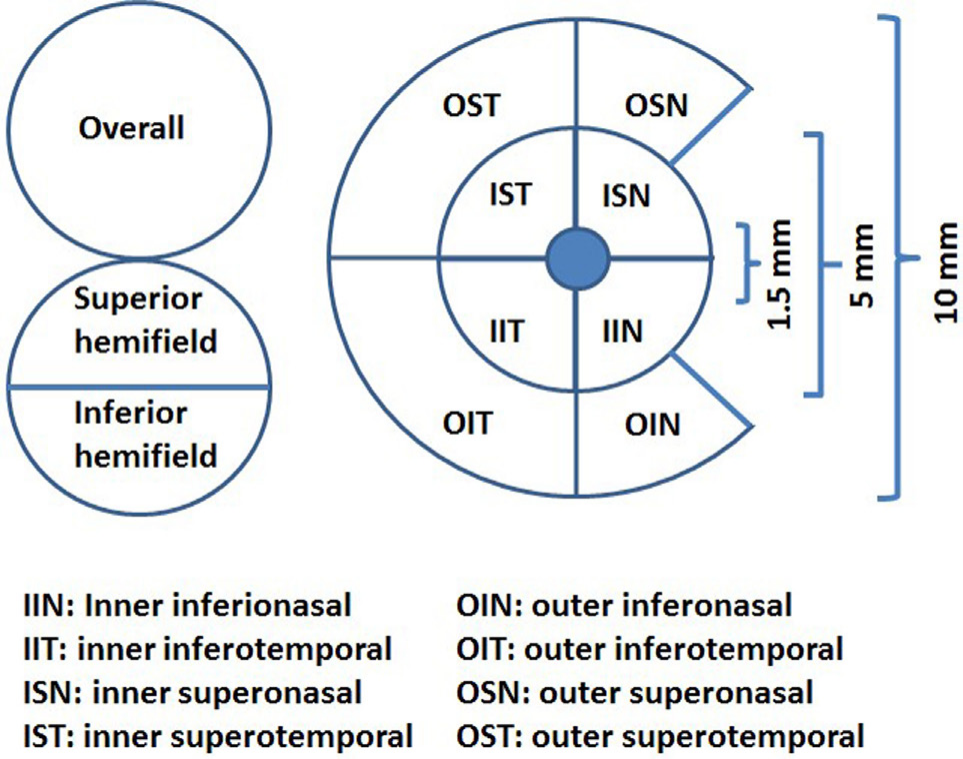
The ganglion cell thickness map as a complex including macular retinal nerve fiber layer, macular ganglion cell layer, and macular inner plexiform layer (ganglion cell complex) (illustration inspired from Ng et al., 2015).

We designed and performed a cross-sectional study measuring the association between the average pRNFL thickness and temporal peripapillary RNFL (T-pRNFL) thickness, macular layer thickness, and demographic and clinical outcomes in MS patients. The macular layers were measured both as GCIP and as a ganglion cell complex (GCC) including macular RNFL, macular ganglion cell layer, and macular inner plexiform layer in an inner ring (with an inner diameter of 1.5 mm and an outer diameter of 5 mm) and an outer ring (with an inner diameter of 5 mm and an outer diameter of 10 mm), see Figure 1. We aimed at identifying the most sensitive method to successfully differentiate MS subtypes from healthy controls (HCs) and the method with highest association with cognitive and physical disability in MS patients. We complemented the cross-sectional study by a longitudinal investigation in which the predictive value of baseline OCT measurement on trajectory of future physical and cognitive disability was assessed. The design of this study followed the Declaration of Helsinki principles and was approved by the local ethical committee. Informed consents were obtained from all participants.

### Study Population

Multiple sclerosis patients were recruited consecutively when coming for their normal routine examination between May 2013 and 2016 to the Neurology Clinic, Neuro Centrum at Karolinska University Hospital, Solna, Sweden. Patients with other neurologic or ocular diseases that could influence the outcome measures were excluded, such as retinal and optic nerve disorders. Patients with acute ON the last 6 months before the OCT measurement were excluded. Related medical records were carefully reviewed, including disease duration, EDSS score, MS subtype [relapsing–remitting (RR), secondary progressive (SP), and primary progressing], treatment duration, and history of ON. Data from the most prior visit to the OCT examination were retrieved. Age was recorded at the time of the visit for OCT examination. We also recruited unaffected controls with the same exclusion criteria. A recently completed study, which compared the repeatability of a Canon OCT HS-100 and a Zeiss Cirrus HD-OCT, also contributed with normal data to the control group of this study (25). Those examinations were performed by an equally experienced operator and with the same OCT instrument as used for examination of the subjects in this study. Controls were selected to age and sex match the MS group.

### Clinical Measurements

Physical disability was assessed by the EDSS and the MS Impact Scale 29 (MSIS-29) physical domain, a patient-reported outcome measure. Cognitive impairment was assessed using Symbol Digit Modalities Test (SDMT) and MSIS-29 psychological domain. All measurements were performed during patients’ routine clinic visits and within an average of 1-month range from the date of OCT examination. Consecutive data from the date of OCT examinations on SDMT, MSIS-29, and EDSS scores was retrieved from the Swedish MS register.

### Spectral Domain OCT

All subjects underwent SD-OCT examination using the Canon OCT HS-100 with software version 1.00 (Canon Europe, Amstelveen, Netherlands) which performs up to 70,000 A-scans/second with an axial resolution of 3 µm. The fixation target was a 2 mm wide cross, and the scan mode “Disk 3D” was used. The site for the “Disk 3D” was 6 mm × 6 mm, and the scan was 512 × 256. From the “Disk 3D” measurement, the average pRNFL thickness for 360° around the optic disk and the temporal portion of the pRNFL (T-pRNFL) were used. For macular measurements, the scan mode “Glaucoma 3D” was used to measure the GCIP thickness over a 10 mm × 10 mm area centered on the fovea. This 3D scan mode is built up of 128 B-scans each consisting of 1,024 A-scans. The total GCIP thickness area consisted of an inner ring (inner center) for macular GCIP thickness and an outer ring for perimacular GCIP thickness. The area consists of eight subfields, four in each ring, see Figure 1. The incorporated algorithm also reports the GCIP thickness for the overall average and the superior and inferior hemi fields of the whole measured area. Measurements were obtained from both eyes of each patient. Only scans without artifacts, signal strength of ≥7 (maximum, 10) and in agreement with the OSCAR-1B criteria (26, 27) were selected for further analysis. The Advised Protocol for OCT Study Terminology and Elements recommendations (APOSTEL recommendation) were followed (28). To reach reproducibility and reliability, the same examiner performed all the measurements on both the control and the MS group.

### Statistical Analysis

R version 3.3.3 (R Core Team, 2016) and package geepack were used for data analyses (29). Generalized estimating equations (GEEs) models were used to account for within-patient inter-eye correlations. We started by comparing the average pRNFL thickness, T-pRNFL thickness, inner and outer GCC thickness, and inner and outer GCIP thickness between MS including all subtypes, each MS subtype separately, and HCs while controlling for the potential confounding effects of age, sex, and previous history of ON. OCT measures with the ability to significantly differentiate MS subtypes with highest regression coefficient were considered optimal.

Similar models were fitted when investigating the association between cognitive and physical disability and OCT measurements at the time of OCT examination (baseline). These models were adjusted for age, sex, disease duration, MS subtype, previous history of ON and duration of exposure to first-line (interferon beta 1a and 1b and glatiramer acetate) and second-line (natalizumab, fingolimod, and rituximab) disease modifying treatments (DMTs). When investigating SDMT score additional adjustment for the number of previous SDMT examinations was made. Since the impact of ON can be significantly different between individuals, we ran the same analyses on eyes with no history of ON to make sure that the statistical adjustment for ON has been adequate.

Finally, we examined the predictive value of baseline average pRNFL, T-pRNFL, inner GCC, and average inner GCIP measures on the trajectory of consecutive SDMT and EDSS scores over 36 months of follow-up. Measurements were categorized into two groups of “reduced” and “normal.” “Normal” OCT measure was defined if the measurement was within one standard deviation of the respective measure in HCs. The OCT measure was considered “reduced” if the measurement was two or more standard deviations lower than the respective measure in HCs. The longitudinal assessment was performed using GEE models adjusted for age, sex, onset age, MS subtype, previous history of ON, and duration of exposure to first-line and second-line DMTs. This was an exploratory analysis so that no correction for multiple comparisons was made.

## Results

Data from examinations of 546 MS patients and 175 HCs were gathered (Table 1). 19 patients were diagnosed with primary progressive multiple sclerosis (PPMS), 391 had relapsing–remitting multiple sclerosis (RRMS), and 136 had converted to secondary progressive multiple sclerosis (SPMS) at the time of OCT examination. 70% of MS patients and 76% HCs were female. As expected, physical disability as measured by the EDSS score was significantly higher in PPMS and SPMS patients compared with RRMS (*P*-value < 0.001). Similarly, PPMS and SPMS patients showed significantly lower SDMT score compared with RRMS patients (*P*-value < 0.001). Nineteen percent of RRMS and 15% of SPMS patients had previous history of ON in one eye. 7% of RRMS and 4% of SPMS patients had history of ON in both eyes.

**Table 1:** Demographic and clinical characteristics of 546 MS patients and 175 HCs.

	HCs (*n* = 175)	PPMS (*n* = 19)	RRMS (*n* = 391)	SPMS (*n* = 136)
Age (mean ± SD)	42.5 ± 15.4	51.8 ± 13.9	38.8 ± 9.5	54.7 ± 10.1
Sex (% female)	76.6	57.9	70.8	66.9
Disease duration (mean years ± SD)	–	11.2 ± 7.9	8.8 ± 7.1	22.9 ± 10
Treatment duration 1st line (mean years ± SD)	–	2.3 ± 4.7	2.7 ± 3.7	5.5 ± 6.1
Treatment duration 2nd line (mean years ± SD)	–	0.3 ± 0.6	1.3 ± 2.0	1.2 ± 2.3
EDSS at baseline [median (IQR)]	–	6 (2)	2 (1.5)	5.5 (2.5)
SDMT at baseline [median (IQR)]	–	50 (11)	57 (15)	47 (27)
MS Impact Scale 29 at baseline [mean (±SD)]				
Physical scale		2.8 ± 0.8	1.7 ± 0.8	2.7 ± 0.9
Psychological scale	–	2.4 ± 0.9	2.1 ± 0.9	2.5 ± 0.9
Previous history of ON in one eye (%)	–	0	18.8	15.4
Previous history of ON in both eyes (%)	–	0	6.6	4.4

*HCs, healthy controls; RRMS, relapsing–remitting multiple sclerosis; SPMS, secondary progressive multiple sclerosis; PPMS, primary progressive multiple sclerosis; ON, optic neuritis; EDSS, Expanded Disability Status Scale; SDMT, Symbol Digit Modalities Test; MSIS, Multiple Sclerosis Impact Scale; DMTs, disease modifying treatments*.

*First-line DMTs include the following: interferon beta 1a and 1b and glatiramer acetate. Second-line DMTs include the following: natalizumab, fingolimod, and rituximab*.

### Comparison of OCT Measures between MS Patients and HCs

The average pRNFL thickness was significantly reduced in both MS with all subtypes included and in all MS subtypes separately compared with HCs (Table 2). However, the T-pRNFL and GCC measurements showed higher sensitivity and superior performance with higher regression coefficient and stronger *P*-values in differentiating MS subtypes from HCs (Table 2). The absolute OCT thickness values of each group are presented in the Table S1 in Supplementary Material.

**Table 2:** Average reduction of optical coherence tomography measures in 19 primary progressive, 391 relapsing–remitting (RR), and 136 secondary progressive (SP) multiple sclerosis (MS) patients compared with 175 healthy controls (HCs).

	Average pRNFL thickness (μm)		T-pRNFL thickness (μm)		Inner GCC thickness (μm)		Inner GCIP thickness (μm)	
HCs	Ref.	*P*-value	Ref.	*P*-value	Ref.	*P*-value	Ref.	*P*-value
MS including all subtypes	−7.58	<0.001	−9.35	<0.001	−8.24	<0.001	−6.31	<0.001
Primary progressive	−9.18	0.009	−16.31	<0.001	−12.16	<0.001	−7.86	<0.001
RR	−6.29	<0.001	−7.74	<0.001	−6.38	<0.001	−4.97	<0.001
SP	−11.29	<0.001	−13.07	<0.001	−12.79	<0.001	−9.81	<0.001

*pRNFL, peripapillary retinal nerve fiber layer; T-pRNFL, temporal peripapillary retinal nerve fiber layer; GCC, ganglion cell complex (composed of macular retinal nerve fiber layer, ganglion cell layer, and inner plexiform layer); GCIP, ganglion cell–inner plexiform layer*.

*Coefficient from generalized estimation equation models adjusted for age, sex, and previous history of optic neuritis*.

### Association between OCT Measures and Cognitive and Physical Disability

Higher EDSS scores were associated with a significant reduction in all the four OCT measures after controlling for potential confounders including MS subtype, sex, age, disease duration, previous ON history, and duration of exposure to first- and second-line DMTs. The most noticeable reduction was seen in inner GCC thickness (Coef: −1.78 μm, *P*-value < 0.001) followed by T-pRNFL thickness (Coef: −1.52 μm, *P*-value < 0.001), and inner GCIP thickness (Coef: −1.28 μm, *P*-value < 0.001). Cognitive performance as measured by SDMT score was also associated with reduction in all OCT measures. None of the OCT measures were associated with MSIS-29 physical and psychological scales. Limiting the analysis to non-ON eyes did not significantly influence the results (Table 3).

**Table 3:** The association between optical coherence tomography measures and cognitive and physical disability at baseline.

	Average pRNFL thickness (μm)		T-pRNFL thickness (μm)		Inner GCC thickness (μm)		Inner GCIP thickness (μm)	
**All eyes [models are adjusted for previous history of optic neuritis (ON)]**								
EDSS score	−1.08	*P* < 0.001	−1.27	*P* < 0.001	−1.77	*P* < 0.001	−1.32	*P* < 0.001
SDMT score	0.14	*P* = 0.001	0.16	*P* < 0.001	0.11	*P* = 0.02	0.07	*P* = 0.04
MSIS physical scale	−0.34	*P* = 0.64	0.15	*P* = 0.83	−1.03	*P* = 0.16	−0.88	*P* = 0.11
MSIS psychological scale	0.16	*P* = 0.81	0.55	*P* = 0.37	−0.59	*P* = 0.36	−0.66	*P* = 0.17

**In eyes with no history of ON**								
EDSS score	−1.06	*P* = 0.006	−1.52	*P* < 0.001	−1.78	*P* < 0.001	−1.28	*P* < 0.001
SDMT score	0.14	*P* = 0.001	0.17	*P* < 0.001	0.12	*P* = 0.02	0.08	*P* = 0.05

*pRNFL, peripapillary retinal nerve fiber layer; T-pRNFL, temporal peripapillary retinal nerve fiber layer; GCC, ganglion cell complex (composed of macular retinal nerve fiber layer, ganglion cell layer, and inner plexiform layer); GCIP, ganglion cell–inner plexiform layer; EDSS, Expanded Disability Status Scale; SDMT, Symbol Digit Modalities Test; MSIS, Multiple Sclerosis Impact Scale*.

*Coefficients from generalized estimating equations models adjusted for multiple sclerosis subtype, sex, age, disease duration, and duration of exposure to first- and second-line disease modifying treatments*.

### Baseline OCT Measures and Future Disability Worsening

**Figure 2:**
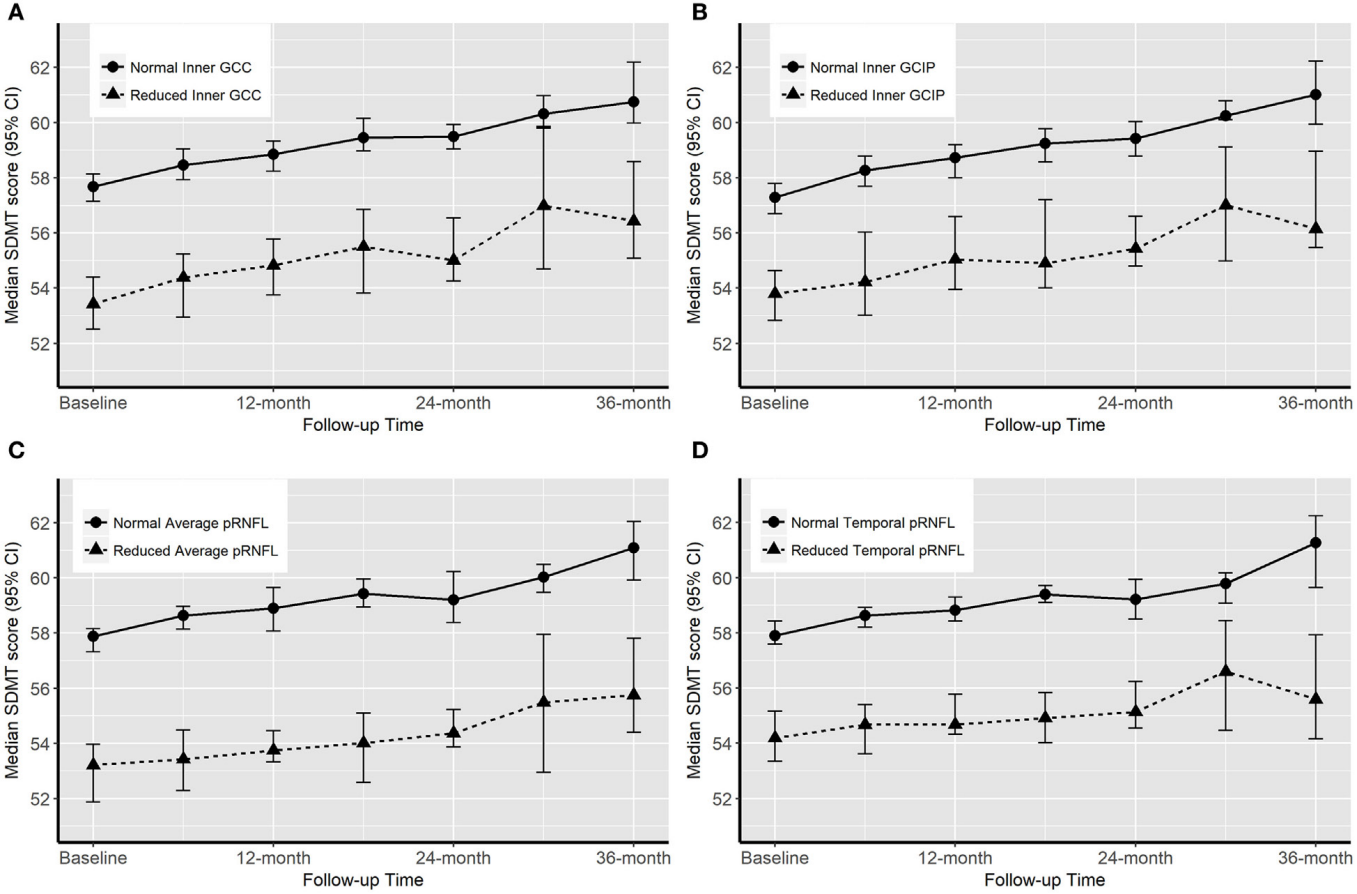
Progression of cognitive impairment [Symbol Digit Modalities Test (SDMT) score] according to the baseline optical coherence tomography (OCT) measures. “Normal” OCT measure falls within one SD of the respective measure in healthy controls (HCs). “Reduced” measures are two or more SDs lower than the respective measure in HCs.

**Figure 3:**
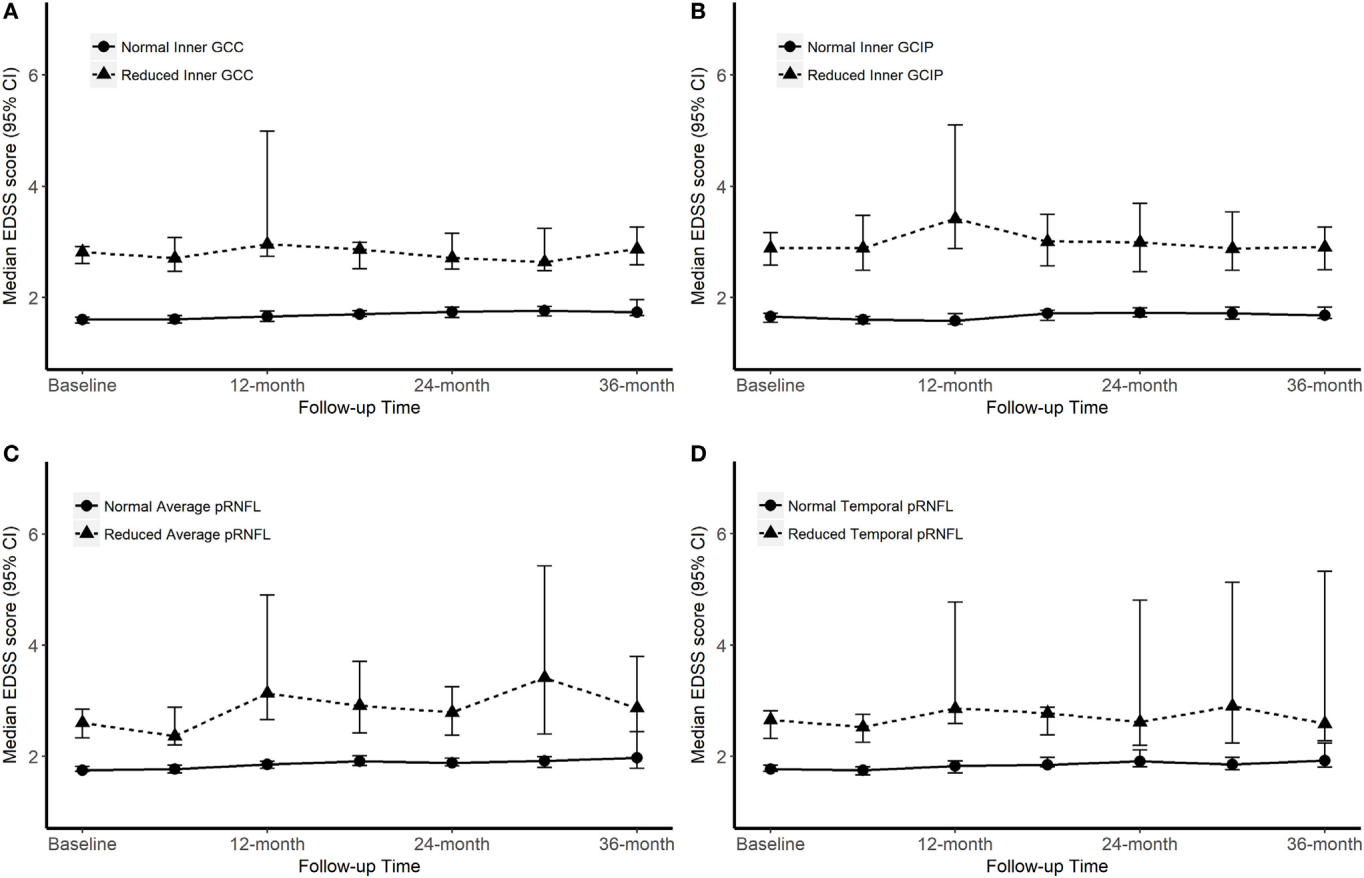
Progression of Expanded Disability Status Scale (EDSS) score according to the baseline optical coherence tomography (OCT) measures. “Normal” OCT measure falls within one SD of the respective measure in healthy controls (HCs). “Reduced” measures are two or more SDs lower than the respective measure in HCs.

Over the follow-up time (up to 36 months), on 6-month average, SDMT increased by 1.2 (*P*-value = 0.005) score. The EDSS score increased by 6-month average of 0.06 score, however, the increase was not significant (*P*-value = 0.2). Patients with reduced OCT measure scored consistently almost 4.4 SDMT (4.1–4.9) score lower than patients with normal OCT measures over the follow-up time. The EDSS score was also consistently remaining higher in patients with reduced OCT measures. Overall, patients with reduced or normal baseline OCT measures did not diverge significantly differently (having similar rate of change) in their future cognitive performance and physical disability worsening (Table 4; Figures 2 and 3).

**Table 4:** Coefficients of interaction between follow-up time and baseline inner GCIP and temporal pRNFL thickness.

	SDMT score		EDSS score	
**Average pRNFL thickness (μm)**				
Normal	Ref.		Ref.	
Reduced	−4.9	*P* = 0.007	0.58	*P* < 0.001
**T-pRNFL thickness (μm)**				
Normal	Ref.		Ref.	
Reduced	−4.1	*P* = 0.02	0.51	*P* = 0.002
**Inner GCC thickness (μm)**				
Normal	Ref.		Ref.	
Reduced	−4.5	*P* = 0.005	0.84	*P* < 0.001
**Inner GCIP thickness (μm)**				
Normal	Ref.		Ref.	
Reduced	−4.1	*P* = 0.01	0.87	*P* < 0.001

*pRNFL, peripapillary retinal nerve fiber layer; T-pRNFL, temporal peripapillary retinal nerve fiber layer; GCC, ganglion cell complex (composed of macular retinal nerve fiber layer, ganglion cell layer, and inner plexiform layer); GCIP, ganglion cell–inner plexiform layer; EDSS, Expanded Disability Status Scale; SDMT, Symbol Digit Modalities Test*.

*Coefficients from GEE models adjusted for multiple sclerosis subtype, sex, onset age, disease duration, baseline SDMT/EDSS score, previous history of optic neuritis, and duration of exposure to first- and second-line DMTs*.

## Discussion

The results of this study with a large OCT data set from a well-defined MS cohort confirm previous findings of reduced GCIP and pRNFL thickness in patients with MS (17, 30, 31). In our study, the T-pRNFL measurement was the most associative measure for both the physical and cognitive disability assessed by EDSS and SDMT, respectively. All included OCT measures showed a difference between MS subtypes, including PPMS patients, and HCs. Many studies have used the average pRNFL thickness as the main outcome parameter, which according to our findings, is not as sensitive as T-pRNFL to reflect the neurodegenerative aspect of MS. The average pRNFL and T-pRNFL were significantly thinner in all MS subtypes. However, the degree of association (as measured by regression coefficient) for the T-pRNFL was higher than pRNFL in all MS subtypes. The average pRNFL was less sensitive than T-pRNFL in the PPMS group (*P* = 0.009). The largest atrophy of the inner retinal layers was seen in the T-pRNFL of PPMS patients (16.31 µm). Previous studies have reported that the RNFL thickness is reduced in eyes without previous ON and is associated with optic radiation damage (32, 33). Klistorner and coworkers (33) showed that the largest thinning was seen in the T-pRNFL when comparing MS eyes without history of ON and controls. These results are in line with earlier studies (4, 34, 35) and our study. A preferential loss of axons in the temporal quadrant of the pRNFL might be that this region subserves the central vision and consists primarily of the small parvocellular axons, which are likely to be more susceptible to damage in MS than the larger sized magnocellular axons (36).

The complex of inner GCC and inner GCIP was significantly reduced in thickness in all MS subtypes, especially the inner GCC in the progressive subtypes (12.16 µm in PPMS and 12.79 µm in SPMS). Interestingly, in one of the most recent studies (37) it was presented that a loss of macular ganglion cells was not accompanied by a pRNFL thinning in newly diagnosed RRMS patients without previous history of ON, compared with HCs. They concluded that this finding suggests that the retinal damage might begin in the macular ganglion cell layer and is then spread to the axons.

In a cross-sectional study by Coric et al. (14), an association between atrophy of the inner retinal layers and cognitive impairment was found. The association was confirmed both with T-pRNFL and GCIP thickness. It was noticeable that the GCIP parameter correlated with all the cognitive tests while the T-pRNFL parameter only correlated with SDMT and a test measuring working memory (memory comparison test). When looking at the average pRNFL and T-pRNFL in our study, a strong and statistically significant correlation with SDMT was found, regardless of history of ON. A statistically significant correlation with SDMT was also found in the inner GCC and inner GCIP; however, this relationship showed a weaker *P*-value. Therefore, the peripapillary OCT measurements seem to reflect MS related cognitive disability better than the macular measurements.

In addition to the association between cognitive dysfunction and inner retinal nerve layer thicknesses, we also found a correlation with physical disability as measured by the EDSS score. Regarding EDSS, all OCT parameters showed a strong and significant correlation (*P* < 0.001), and it was not possible to conclude if any measurement better explained physical disability than the others.

Garcia-Martin et al. (38) recently reported an association between reduced quality of life (based on 54-item Multiple Sclerosis Quality of Life Scale Score) and peripapillary RNFL thinning in the temporal quadrant over 5 years. In this study, another patient-reported outcome measure was used (MSIS-29), and no correlation was found with any of the inner retinal layer OCT measurements.

Some attempts have been made to study the possibility to use inner retinal thickness parameters to predict the future cognitive and/or physical disability worsening. Coric and coworkers (14) reached the conclusion that atrophy of the inner retinal layers is significantly associated with increased odds of being cognitively impaired. A similar idea was tested by Martinez-Lapiscina et al. (10), although with focus on physical disability. Patients with an average pRNFL equal to or below a certain cutoff value had double risk of disability worsening at any point after 1–3 years. Graham et al. showed that progressive losses of retinal axons were more apparent on T-pRNFL than on GCIP thickness over a 3-year follow-up including RRMS patients (17). To investigate the predictive value of baseline OCT measurements we looked at patients’ disability worsening over a 36-month time period. Overall, while groups with the normal and reduced OCT measures were significantly different in their level of physical and cognitive disability, both groups’ trajectories of disability and impairment worsening were almost parallel over the 36-month time period. Therefore, the longitudinal results from this study cannot confirm the value of patients’ baseline OCT measures in predicting the future physical and cognitive disability.

It has been suggested that the retinal degeneration measured as GCIP or pRNFL could serve as an estimate of the global brain atrophy seen in MS, and that OCT is a useful tool for clinical monitoring and as outcome in investigative trials (8, 12, 39, 40). Compared with magnetic resonance imaging (MRI) OCT is a less time consuming, more cost-efficient, and simpler technique. It is also an imaging technique that is specific for axonal loss and might be a complement to MRI for MS.

From a clinical perspective, it might be preferable to measure neuronal changes in the macula rather than in the optic disk. The OCT images are more easily acquired when the patient fixates straight ahead (macula scan). It is also known that the repeatability is better in the macula than in the optic disk (25). On the other hand, macular images are influenced by common retinal abnormalities, such as epiretinal membrane and age-related macular degeneration. However, MS is a disease that primarily affects the younger population so the risk of having any age-related changes in the macula is small.

The strength of this study, except the large sample size, is the identification of OCT parameters that correlates strongly with both cognitive impairment and physical disability in MS. A limitation with the study and a potential bias is that we did not have the same number of patients through the whole 36-month time period. Therefore, investigations regarding OCT outcomes over time are ongoing.

In conclusion, our results suggest that the T-pRNFL is the most important OCT estimate and that OCT might be a valuable tool in assessing neuronal and axonal degeneration in MS. This study has a cross-sectional value demonstrating a strong association for T-pRNFL with both cognitive and physical disability; however, it is not clear if T-pRNFL can serve as a risk indicator to predict disability worsening.

## Ethics Statement

This study was carried out in accordance with the recommendations of Etiska Regionala etikprövningsnämnden with written informed consent from all subjects. All subjects gave written informed consent in accordance with the Declaration of Helsinki. The protocol was approved by the Etiska Regionala etikprövningsnämnden.

## Author Contributions

UB, AM, RB, and MN—substantial contributions to the design, acquisition, analysis, and interpretation of data and drafting of the manuscript. MH—substantial contributions to the acquisition, analysis, and interpretation of data and critical revision for intellectual content. JH and FP—substantial contributions to the design, acquisition, analysis, and interpretation of data and critical revision for intellectual content. TO, IK, LB, OZ, and MW-R—substantial contributions to the analysis and interpretation of data and critical revision for intellectual content. All the authors gave final approval of the version to be published and were agreed to be accountable for all aspects of the manuscript in ensuring that questions related to the accuracy or integrity of any part of the work are appropriately investigated and resolved.

## Conflict of Interest Statement

The authors declare that the research was conducted in the absence of any commercial or financial relationships that could be construed as a potential conflict of interest.

## Abbreviations

T-pRNFL, temporal peripapillary retinal nerve fiber layer; pRNFL, peripapillary retinal nerve fiber layer; GCIP, ganglion cell–inner plexiform layer; GCC, ganglion cell complex (composed of macular retinal nerve fiber layer, macular ganglion cell layer, and macular inner plexiform layer).
