# Supplementary material for: The Temporal Retinal Nerve Fiber Layer Thickness Is the Most Important Optical Coherence Tomography Estimate in Multiple Sclerosis
Source: Front Neurol. 2017 Dec 13;8:675. doi: 10.3389/fneur.2017.00675 (PMC5733353; doi:10.3389/fneur.2017.00675)
Supplement: Supplementary file 1 [file data_sheet_1.docx]

**SUPPLEMENTARY TABLE 1.** Mean values of the retinal layer thickness measurements by OCT for MS patients and healthy controls.

|  | **Average pRNFL (µm)** | **tRNFL (µm)** | | **Inner GCC (µm)** | **Outer GCC (µm)** | **Inner GCIP (µm)** | **Outer GCIP (µm)** |
| --- | --- | --- | --- | --- | --- | --- | --- |
| **Healthy Controls** | 100.1 (SD 9.7) | | 71.8 (SD 11.0) | 107.2 (SD 8.2) | 91.4 (SD 7.3) | 77.2 (SD 6.2) | 44.8 (SD 3.8) |
| **MS including all subtypes** | 89.7 (SD 14.2) | | 60.3 (SD 14.3) | 97.3 (SD 13.0) | 88.0 (SD 8.4) | 70.0 (SD 9.5) | 44.0 (SD 3.8) |
| **Primary Progressive** | 89.9 (SD 16.0) | | 56.4 (SD 14.6) | 95.2 (SD 13.6) | 87.8 (SD 8.4) | 69.3 (SD 9.9) | 44.2 (SD 3.5) |
| **Relapsing Remitting** | 91.1 (SD 14.2) | | 61.6 (SD 14.2) | 99.2 (SD 12.8) | 89.1 (SD 8.2) | 71.6 (SD 9.2) | 44.7 (SD 3.7) |
| **Secondary Progressive** | 85.5 (SD 13.3) | | 57.1 (SD 13.8) | 91.9 (SD 12.1) | 84.8 (SD 8.3) | 65.5 (SD 8.6) | 42.0 (SD 3.3) |

pRNFL: peripapillary Retinal Nerve Fiber Layer, tRNFL: temporal peripapillary Retinal Nerve Fiber Layer, GCC: Ganglion Cell Complex (composed of macular retinal nerve fiber layer, ganglion cell layer and inner plexiform layer), GCIP: Ganglion Cell-Inner Plexiform layer, SD = Standard Deviation.
